# Modification of additive effect between vitamins and ETS on childhood asthma risk according to *GSTP1* polymorphism : a cross -sectional study

**DOI:** 10.1186/s12890-015-0093-0

**Published:** 2015-10-22

**Authors:** So-Yeon Lee, Bong-Seong Kim, Sung-Ok Kwon, Se-Young Oh, Hye Lim Shin, Young-Ho Jung, Eun Lee, Song-I Yang, Hyung Young Kim, Ju-Hee Seo, Hyo-Bin Kim, Ji-Won Kwon, Hae-Ran Lee, Soo-Jong Hong

**Affiliations:** Department of Pediatrics, Hallym Sacred Heart Hospital, Hallym University College of Medicine, 39, Gwanpyeong-ro 138 beon-gil, Dongan-gu, Anyang, Gyeonggido 431-828 South Korea; Department of Pediatrics, Gangneung Asan Hospital, University of Ulsan College of Medicine, Gangneung, South Korea; Department of Food and Nutrition, College of Human Ecology, Kyung Hee University, Seoul, South Korea; Research Center for Standardization of Allergic Diseases, Asan Institute for Life Sciences, University of Ulsan College of Medicine, Seoul, South Korea; Department of Pediatrics, CHA Bundang Medical Center, CHA University School of Medicine, Yatap-dong Bundang-gu, Seongnam, Gyeonggido 463-712 South Korea; Department of Pediatrics, Childhood Asthma Atopy Center, Asan Medical Center, University of Ulsan College of Medicine, 88 Olympic-ro 43-gil, Songpa-gu, Seoul 138-736 South Korea; Department of Pediatrics, Kosin University Gospel Hospital, Busan, South Korea; Department of Pediatrics, Korea Cancer Center Hospital, Seoul, South Korea; Department of Pediatrics, Sanggye Paik Hospital, Inje University College of Medicine, Seoul, South Korea; Department of Pediatrics, Seoul National University Bundang Hospital, Seongnam, South Korea

**Keywords:** Asthma, Vitamin A, Antioxidant, Environmental tobacco smoke, Oxidative stress, Polymorphism

## Abstract

**Background:**

Asthma is characterized by airway inflammation, and bronchial airways are particularly susceptible to oxidant-induced tissue damage.

**Objective:**

To investigate the effect of dietary antioxidant intake and environmental tobacco smoke (ETS) on the risk of childhood asthma according to genotypes susceptible to airway diseases.

**Methods:**

This cross-sectional study included 1124 elementary school children aged 7–12 years old. Asthma symptoms and smoking history were measured using the International Study of Asthma and Allergies in Childhood (ISAAC) questionnaire. Intake of vitamin A (including retinol and β-carotene), C, and E was measured by a semi-quantitative food frequency questionnaire (FFQ). *GSTP1* polymorphisms were genotyped from peripheral blood samples.

**Results:**

ETS was significantly associated with presence of asthma symptoms (adjusted odds ratio [aOR], 2.48; 95 % confidence interval [CI], 1.29–4.76) and diagnosis (aOR, 1.91; 95 % CI, 1.19–3.06). Dietary antioxidant intake was not associated with asthma symptoms, although ETS plus low vitamin A intake showed a significant positive association with asthma diagnosis (aOR, 2.23; 95 % CI, 1.10–4.54). Children with AA at nucleotide 1695 in *GSTP1* who had been exposed to ETS and a low vitamin A intake have an increased risk of asthma diagnosis (aOR, 4.44; 95 % CI,1.58–12.52) compared with children who had not been exposed to the two risk factors. However, ETS exposure and low vitamin A intake did not significantly increase odds of asthma diagnosis in children with AG or GG genotypes.

**Conclusion:**

Low vitamin A intake and ETS exposure may increase oxidative stress and thereby risk for childhood asthma. These relationships may be modified by gene susceptibility alleles of *GSTP1*.

**Electronic supplementary material:**

The online version of this article (doi:10.1186/s12890-015-0093-0) contains supplementary material, which is available to authorized users.

## Background

Asthma is a chronic inflammatory disease of the respiratory tract, and the bronchial airways are particularly susceptible to oxidation-induced tissue damage [1]. Environmental tobacco smoke (ETS) worsens asthma symptoms and leads to poor asthma control in adults and children [2, 3]. Current guidelines for asthma treatment recommend avoiding exposure to tobacco smoke, including both active smoking and ETS [4]. Children who are exposed to ETS in their homes have lower peak lung function than those who are not exposed [5].

Oxidative stress results in inflammation and tissue damage in the respiratory system and, subsequently, immune system damage. Indeed, individuals with lower cellular reducing capacity have an increased risk of developing asthma [6]. Tobacco smoke is a key source of free radicals related to oxidative stress and therefore increases the risk for asthma [7, 8].

Antioxidants provide protection by preventing oxidative DNA damage [9]. A deficiency in dietary antioxidants is associated with increased asthma risk by increasing susceptibility to oxidative stress [10, 11]. The combination of smoking and low antioxidant levels increase free radicals and therefore lead to antioxidant depletion and oxidative stress [12–14]. Smoking is strongly associated with reduced blood concentration of vitamin C, as well as α- and β-carotene [15]. Smoking has also been shown to deplete endogenous antioxidants such as vitamins C and E, β-carotene, ubiquinol, glutathione, and α-lipoic acid [16]. Antioxidant depletion increases individual vulnerability to free radicals and other oxidant species produced by tobacco smoking, and thus increases morbidity, the rate of aging, and risk of death.

Dietary supplementation with antioxidants may reduce the overall oxidative burden that is increased by cigarette smoking [16]. Previous studies indicate that consumption of carotenoids, a group of dietary antioxidants, may reduce the smoking-induced increase in colorectal cancer risk [17]. Another study shows that the intake of nutrients with antioxidant properties may reduce lung function decline in older adults exposed to cigarette smoke [18]. Enhancing antioxidant defenses may reduce the cumulative effects of oxidative damage and perhaps the risk of developing childhood asthma. To the best of our knowledge, no research has evaluated the impact of ETS and dietary antioxidants on the risk of childhood asthma.

Genetic factors, such as gene polymorphisms that alter the antioxidant response, may also contribute to the relationship between asthma and ETS or deficiency of dietary antioxidants. Glutathione S-transferases (GSTs) are a group of enzymes that are reported to neutralize the effects of tobacco smoke and reduce oxidative stress [19]. Most studies investigating genetically associated susceptibility to respiratory abnormalities have focused on *GSPT1*, the most abundant GST isozyme in the lungs, as well as *GSTM1* and *GSTT1*. The functional sequence variant in *GSTP1* at codon 105 (Ile105Val -rs1695) has been associated with asthma in some [20, 21] but not all studies [22]. This variant has been shown to protect against and increase the risk of asthma. Several studies report that *GSTP1* genotypes modulate the effect of environment-induced respiratory symptoms and asthma in children [23, 24]. However, the effect of gene polymorphisms on the response to dietary antioxidants and ETS, and subsequently asthma risk, is poorly understood.

We investigated the effect of dietary antioxidant intake and ETS on the presence of asthma symptoms according to *GSTP1* polymorphism in children age 7–12 years.

## Methods

### Study population

All children attending a single elementary school in Seoul, Korea were invited to participate in this study (total 1376 individuals age 7–12 years). Of these, 1356 responded to the questionnaire (response rate 98.6 %). Of the respondents, 1111 children (577 boys and 529 girls) were included in this study. The 245 children who did not answer the food questionnaire were excluded due to insufficient information about their calorie intake. The parents or guardians of all participants signed a written informed consent form. This study was approved by the International Review Board of Asan Medical Center, University of Ulsan.

### Questionnaire survey

A Korean version of the International Study of Asthma and Allergies in Childhood (ISAAC) questionnaire and the food frequency questionnaire (FFQ) were completed by each participant’s parents or guardians. The modified Korean version of ISAAC has been validated for assessment of allergy symptoms and diagnosis of allergic diseases in Korean children [25]. Definitions of asthma were based on answers to specific questions, such as “Ever had wheezing?”, “Ever had a doctor’s diagnosis of asthma?”, and “Ever had wheezing during the last 12 months?” [25].

The parents or guardians of each subject were given a questionnaire to complete, which included questions on social background, family history of allergic diseases, and smoking histories. A participant was considered to have exposure to ETS if the answer to the question “Has your child ever been exposed to smoke from tobacco more than once a week?” was “yes.” [26] Frequency of ETS was determined by the answer to the question “If your child was exposed to tobacco smoke more than once a week, how many times was your child exposed to tobacco smoke for a week?”

Dietary intake was assessed by the semi-quantitative FFQ. This questionnaire has been validated previously and includes 113 food items with nine non-overlapping intake frequencies over the preceding year (ranging from “rarely eaten” to “eaten more than 3 times per day”) and three portion sizes (small, average, or large) [27]. Using the Computer Aided Nutritional Analysis Program III (CAN PRO III) developed by the Korean Nutrition Society, the amount of each food item included in the FFQ was converted into grams, from which daily nutrient intake was calculated [28]. Dietary intake of the antioxidant micronutrients vitamins A, C, and E, retinol, and carotene were analyzed.

### Serum total IgE levels and pulmonary function test

The concentration of serum total IgE was measured by fluorescent enzyme immunoassay using the AutoCAP System (Phadia AB, Uppsala, Sweden). Data on lung function were collected by trained field technicians who visited the schools. Baseline spirometry was performed for each study participant according to the American Thoracic Society guidelines [29] using a portable microspirometer (Microspiro HI-298; Chest Corporation, Tokyo, Japan). We measured forced vital capacity (FVC), forced expiratory volume in 1 s (FEV_1_), and the mean forced expiratory flow during the middle half of FVC (FEF_25–75%_).

### Genotyping

Genomic DNA was extracted from peripheral blood of study participants. *GSTP1* (rs1695) polymorphisms were genotyped using the TaqMan assay, assay C_3237198_20 (ABI, Foster City, CA, USA) according to the manufacturer’s instructions. End-point readings were obtained using an ABI PRISM 7900 HT Sequence Detection System (ABI).

### Statistical analysis

Nutrients were adjusted to total energy intake using the residual method. Adjusted odds ratios (aORs) and 95 % confidence intervals (CIs) were obtained using logistic regression analysis. Statistical analyses were performed using SAS for Windows (version 9.2). Multiple logistic regression analysis was performed by adjusting for key covariates such as age, sex, body mass index (BMI), parental history of allergic diseases, maternal education, log-transformed total energy intake, and monthly household income. A P-value of < 0.05 was considered to indicate statistical significance.

## Results

### Subject characteristics

Descriptive statistics across the total sample are summarized in Additional file 1: Table S1. Parental history of asthma, maternal education, household income, parental smoking, exposure to ETS, and wheeze in previous 12 months were not significantly different between the included and excluded children. However, the included group of children was younger and had a greater proportion of females, lower BMI, and a greater proportion of children with asthma than the excluded group.

The prevalence of children with an asthma diagnosis was 10.3 %, and 5.8 % of the children had experienced wheezing in the previous 12 months (Table 1). Both wheezing within the previous 12 months and an asthma diagnosis were more prevalent in children exposed to ETS than in those who were not exposed (*P* = 0.02 and *P* = 0.04, respectively). Serum IgE level and pulmonary function did not differ between the two groups.

**Table 1:** Study population characteristics^*^

Parameter		Total	ETS (+)	ETS (−)	*P*-value
*n*		1111	403	708	
Age (years)		9.48 ± 1.73	9.54 ± 1.68	9.44 ± 1.76	0.384
Sex (male/female)		577/529	366/337	211/192	0.925
BMI		18.46 ± 3.30	18.77 ± 3.48	18.28 ± 3.18	0.024
Parental history of asthma		38/875 (4.3 %)	14/315(4.4 %)	24/560(4.3 %)	0.912
Parental history of AR		347/885 (39.2 %)	109/322(33.9 %)	238/563(42.3 %)	0.014
Parental history of AD		88/896 (9.8 %)	28/323(8.7 %)	60/573(10.5 %)	0.384
Maternal education	Low (≤ high school)	391/1090 (35.9 %)	162/391(41.4 %)	229/699(32.8 %)	0.004
	High (> high school)	699/1090 (64.1 %)	229/391(58.6 %)	470/699(67.2 %)	
Household income (10,000 Korean won)	≤299	312/1055 (29.6 %)	134/386(34.7 %)	178/669(26.6 %)	0.019
	300–399	292/1055 (27.7 %)	102/386(26.4 %)	190/669(28.4 %)	
	≥400	451/1055 (42.8 %)	150/386(38.9 %)	301/669(45.0 %)	
Paternal smoking	Non-smoker	293/1078 (27.2 %)	34/394(8.6 %)	259/684(37.9 %)	<.0001
	Past smoker	263/1078 (24.4 %)	36/394(9.1 %)	227/684(33.2 %)	
	Current smoker	522/1078 (48.4 %)	324/394(82.2 %)	198/684(29.0 %)	
Maternal smoking	Non-smoker	1075/1091 (98.5 %)	382/391(97.7 %)	693/700(99.0 %)	
	Past smoker	6/1091 (0.6 %)	2/391(0.5 %)	4/700(0.6 %)	0.077
	Current smoker	10/1091 (0.9 %)	7/391(1.8 %)	3/700(64.2 %)	
Frequency of ETS (per week)	≤ twice		85/215 (39.5 %)		
	2–4 times		59/215 (27.4 %)		
	≥5 times		71/215 (33.0 %)		
Serum total IgE (kU/L)*		58.13 ± 4.01	59.45 ± 3.65	57.38 ± 4.22	0.693
Pulmonary function test*	FVC (%)	89.34 ± 1.13	89.35 ± 1.13	89.33 ± 1.14	0.982
	FEV_1_(%)	96.91 ± 1.13	96.86 ± 1.13	96.93 ± 1.13	0.932
	FEV_1_/FVC	92.12 ± 1.07	91.99 ± 1.07	92.2 ± 1.06	0.588
	FEF_25–75%_ (%)	90.63 ± 1.38	91.89 ± 1.31	89.96 ± 1.41	0.554
Wheeze in previous 12 months		62/1069 (5.8 %)	31/386 (8.0 %)	31/683 (4.5 %)	0.019
Asthma diagnosis		110/1064 (10.3 %)	50/390 (12.8 %)	60/674 (8.9 %)	0.043

*AR* allergic rhinitis, *AD* airway disease; *BMI* body mass index, *ETS* environmental tobacco smoke, *FVC* forced vital capacity, *FEV1* forced expiratory volume in 1 s, *FEF*_*25–75%*_ forced expiratory flow during the middle half of FVC

^*^Values are mean ± SD or *n* (%)

A parental history of asthma was identified in 4.28 % of all subjects. Children who were not exposed to ETS tended to have mothers with higher education levels and come from families with higher household incomes than children exposed to ETS (*P* = 0.01 for both).

Daily dietary antioxidant intake is described in Table 2. The average intake of total calories and vitamin C were within the dietary reference intake (DRI) range for Koreans. The average vitamin A and E intake was slightly above the DRI for Koreans. Dietary antioxidant intake did not differ between children exposed to ETS and those who were not exposed.

**Table 2:** Dietary antioxidant intake

	DRIs for Koreans (children aged 6–14 years)	ETS (+)	ETS (−)	*P*-value^*^
Energy (kcal)	1500–1900	1808.31 ± 697.85	1826.91 ± 756.98	0.686
Vitamin A (μg RE)	400–700	712.28 ± 400.25	750.86 ± 479.37	0.151
Retinol (μ)	-	227.88 ± 119.99	221.25 ± 120.26	0.376
Carotene (μg)	-	2719.00 ± 1976.33	2979.41 ± 2487.95	0.055
Vitamin C (mg)	60–100	77.08 ± 62.41	77.05 ± 54.34	0.993
Vitamin E (mg)	7–10	13.40 ± 7.44	13.90 ± 8.46	0.308

*DRIs* dietary reference intakes, *ETS* environmental tobacco smoke

Nutrients were adjusted for total energy intake using the residual method

^*^Nutrient intake in children exposed to ETS exposure versus those not exposed to ETS

### Relationship between dietary antioxidant intake and asthma risk

Multivariate analysis showed no association between intake of any of the dietary antioxidants (vitamins A, C, and E, retinol, and carotene) and asthma diagnosis or wheeze within the previous 12 months (Table 3).

**Table 3:** Associations among antioxidant nutrient intake, environmental tobacco smoke, and prevalence of asthma symptoms

Variable		Wheeze symptom in the previous 12 months		Asthma diagnosis	
		*n* (%)	aOR^f^ (95 % CI)	*n* (%)	aOR^f^ (95 % CI)
Antioxidant intake^h^					
Vitamin A^a^	Low	46/725 (6.34)	1.00	74/720 (10.28)	1.00
	High	17/360 (4.72)	0.53 (0.24, 1.17)	36/362 (9.94)	0.77 (0.44, 1.34)
Retinol^b^	Low	42/722 (5.82)	1.00	59/714 (8.26)	1.00
	High	21/363 (5.79)	0.59 (0.27, 1.28)	51/368 (13.86)	1.51 (0.88, 2.60)
Carotene^c^	Low	41/725 (5.66)	1.00	71/723 (9.82)	1.00
	High	22/360 (6.11)	0.95 (0.46, 2.00)	39/359 (10.86)	0.95 (0.55, 1.62)
Vitamin C^d^	Low	38/725 (5.24)	1.00	66/727 (9.08)	1.00
	High	25/360 (6.94)	0.97 (0.45, 2.07)	44/355 (12.39)	1.14 (0.66, 1.97)
Vitamin E^e^	Low	34/725 (4.69)	1.00	60/726 (8.26)	1.00
	High	29/360 (8.06)	1.67 (0.72, 3.87)	50/356 (14.04)	1.57 (0.85, 2.89)
Environmental tobacco smoke^g^	No	31/683 (4.54)	1.00	60/674 (8.90)	1.00
	Yes	31/386 (8.03)	2.48 (1.29, 4.76)*	50/390 (12.82)	1.91 (1.19, 3.06)*

*aOR* adjusted odds ratio, *BMI* body mass index

^a^Cut-off point for dietary vitamin A, 803.84 μg/day

^b^Cut-off point for dietary retinol, 253.69 μg/day

^c^Cut-off point for dietary β-carotene, 3081.38 μg/day

^d^Cut-off point for dietary vitamin C, 81.4 mg/day

^e^Cut-off point for dietary vitamin E, 14.68 mg/day

^f^aOR: Adjusted by age, sex, BMI (continuous), parental history of asthma, exposure to environmental tobacco smoke, maternal education, household income, and log-transformed total energy intake

^g^aOR: Adjusted for the same confounders as above with the exception of environmental tobacco smoke

^h^Nutrients were adjusted for total energy intake

*P-value < 0.01

### Relationship between ETS and asthma risk

ETS significantly increased the odds of wheeze in the previous 12 months (aOR, 2.48; 95 % CI, 1.29–4.76) and asthma diagnosis (aOR, 1.91; 95 % CI, 1.19–3.06) (Table 3).

### Effects of ETS and dietary antioxidant intake on asthma risk

In a combined analysis of dietary antioxidant intake and ETS, we found an additive effect of a low vitamin A intake and ETS exposure for increasing the risk of asthma diagnosis (Table 4). Among children who were exposed to ETS, those with low dietary intake of vitamin A and retinol had significantly greater odds of reporting wheeze within the previous 12 months than children with high dietary intakes (vitamin A: aOR, 4.43, 95 % CI, 1.51–12.96; retinol: aOR, 5.15, 95 % CI, 1.63–16.25).

**Table 4:** The combined effects of dietary antioxidant intake and environmental tobacco smoke on the risk for asthma

Variable		Wheezing symptoms in previous 12 months					Asthma diagnosis				
		*N* (%)	aOR^a^ (95 % CI)			*P*-value	*N* (%)	aOR^a^ (95 % CI)			*P*-value
Vitamin A	ETS										
High	No	6/148(3.90)	1.00				19/131(12.67)	1.00			
Low	No	14/309(4.33)	1.59	(0.54	4.67)	0.40	24/290(7.64)	0.80	(0.39	1.64)	0.55
High	Yes	6/89(6.32)	2.01	(0.61	6.64)	0.25	11/86(11.34)	0.98	(0.44	2.21)	0.96
Low	Yes	17/62(9.50)	4.43	(1.51	12.96)	<0.01	31/152(16.94)	2.23	(1.10	4.54)	0.03
Retinol	ETS										
High	No	5/149(3.25)	1.00				20/134(12.99)	1.00			
Low	No	15/308(4.64)	2.67	(0.85	8.34)	0.09	23/287(7.42)	0.73	(0.36	1.49)	0.39
High	Yes	8/83(8.79)	4.37	(1.30	14.63)	0.02	19/74(20.43)	2.16	(1.05	4.43)	0.04
Low	Yes	15/168(8.20)	5.15	(1.63	16.25)	0.01	23/164(12.30)	1.29	(0.63	2.66)	0.49
Carotene	ETS										
High	No	7/144(4.64)	1.00				18/127(12.41)	1.00			
Low	No	13/313(3.99)	0.88	(0.31	2.48)	0.81	25/294(7.84)	0.72	(0.36	1.46)	0.37
High	Yes	8/90(8.16)	2.03	(0.69	6.00)	0.20	13/86(13.13)	1.16	(0.53	2.54)	0.71
Low	Yes	15/161(8.52)	2.45	(0.87	6.85)	0.09	29/152(16.02)	1.84	(0.90	3.73)	0.09
Vitamin C	ETS										
High	No	7/153(4.38)	1.00				21/133(13.64)	1.00			
Low	No	13/304(4.10)	1.09	(0.38	3.15)	0.87	22/288(7.10)	0.58	(0.28	1.18)	0.13
High	Yes	9/90(9.09)	2.65	(0.92	7.64)	0.07	14/86(14.00)	1.13	(0.53	2.40)	0.75
Low	Yes	14/161(8.00)	2.61	(0.90	7.59)	0.08	28/152(15.56)	1.55	(0.76	3.13)	0.23
Vitamin E	ETS										
High	No	9/166(5.14)	1.00				23/145(13.69)	1.00			
Low	No	11/291(3.64)	0.78	(0.26	2.31)	0.65	20/276(6.76)	0.56	(0.26	1.21)	0.14
High	Yes	11/84(11.58)	3.32	(1.25	8.80)	0.02	18/81(18.18)	1.67	(0.83	3.36)	0.15
Low	Yes	12/167(6.70)	1.58	(0.54	4.64)	0.41	24/157(13.26)	1.22	(0.57	2.58)	0.61

*ETS* environmental tobacco smoke

^a^aOR: Adjusted for age, sex, BMI (continuous), parental history of asthma, maternal education, household income, and log-transformed total energy intake

### Effect of ETS and dietary antioxidant intake on asthma risk according to *GSTP1* polymorphism

**Fig. 1:**
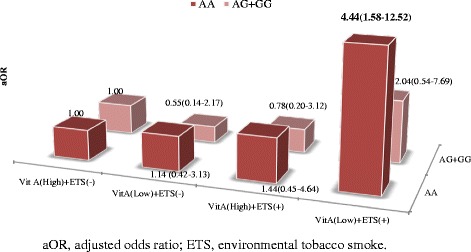
Combined effects of vitamin A and environmental tobacco smoke (ETS) on the risk of asthma diagnosis regarding *glutathione S-transferase P1* (*GSTP1*) polymorphism

**Fig. 2:**
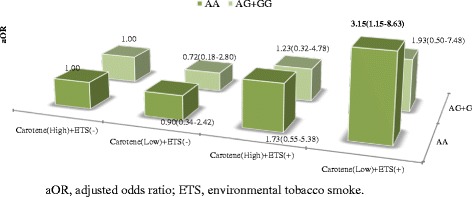
Combined effects of carotene and environmental tobacco smoke (ETS) on the risk of asthma diagnosis regarding *glutathione S-transferase P1* (*GSTP1*) polymorphism

The distribution of *GSTP1* polymorphism was in Hardy–Weinberg equilibrium. The number and proportion of subjects with the AA, AG, and GG *GSTP1* polymorphisms were 597 (62.6 %), 319 (33.4 %), and 38 (4.0 %), respectively. The Hardy-Weinberg equilibrium P-value for this polymorphism was 0.569. The relationship between dietary antioxidant intake and ETS was more apparent in children with AA at nucleotide 1695 of the *GSTP1*. Children with the AA genotype who had been exposed to ETS and had low intakes of vitamin A or carotene were more likely to have an asthma diagnosis than children with no ETS exposure and high intakes of vitamin A or carotene (vitamin A: aOR, 4.44, 95 % CI, 1.58–12.52; carotene: aOR, 3.15, 95 % CI, 1.15–8.63; Additional file 2: Table S2, Figs. 1 and 2). However, low vitamin intake and ETS exposure did not significantly increase the odds of asthma diagnosis in children with AG or GG genotypes.

In children with an AA genotype, children with ETS and a high retinol intake were more likely to have an asthma diagnosis (aOR, 4.18, 95 % CI, 1.51–11.57; Additional file 1: Table S1), although this association was not observed in children with the AG or GG genotypes.

## Discussion

This study showed an additive effect of low dietary intake of vitamin A and ETS exposure for increasing risk for asthma symptoms. Additionally, the AA *GSTP1* polymorphism was associated with an increased risk for asthma in children who were exposed to ETS and had a low dietary intake of vitamin A and carotene. Children who were exposed to ETS were significantly more likely to report wheeze within the previous 12 months and have an asthma diagnosis. Although overall antioxidant intake was not associated with presence of asthma symptoms, children who were exposed to ETS and had a low vitamin A intake were more likely to report asthma symptoms. This trend was particularly notable in children carrying the *GSTP1* genotype AA, which has been associated with an increased risk for asthma. Our study suggested that low vitamin A intake increased susceptibility to development of ETS-associated childhood asthma by decreasing antioxidant capacity, and the oxidative stress-related *GSTP1* gene further modified this association.

Oxidative stress occurs when the generation of oxidant molecules (i.e., free radicals) exceeds the available antioxidant defenses [30]. Inflammatory disorders such as asthma and allergic rhinitis may be mediated by oxidative stress [25], which occurs as a result of endogenous inflammation and following environmental exposure to toxic substances such as cigarette smoke and air pollutants in allergic airway diseases [6]. Exposure to ETS is a major environmental factor that influences the development and aggravation of asthma and impaired lung function in childhood [7].

Cigarette smoke inhalation increases exposure to reactive oxygen species [31] to a level that may overwhelm endogenous antioxidant defenses in asthmatic patients who already have exacerbated levels of oxidative stress [6]. A controlled human exposure model has shown that glutathione levels are reduced in the bronchial and nasal airways following exposure to air pollutants [32]. The body produces numerous antioxidants endogenously, but the quantity is often insufficient to prevent oxidative stress. Exogenous antioxidants, such as dietary nutrients, can supplement the endogenous system to help defend against free radicals. Smoking is associated with reduced circulating concentration of antioxidants in the blood [33]. This may be because dietary and supplemental antioxidant intake tends to be lower in smokers than in non-smokers and because smoke-induced oxidative stress increases the degradation or transformation of circulating antioxidant micronutrients into biologically inactive components [34] or even into pro-oxidants [35]. Therefore, dietary antioxidant intake may influence the relationship between exposure to ETS and the risk of asthma symptoms.

Several studies suggest that low serum levels or dietary intake of antioxidants may be risk factors for asthma [9, 10]. Dietary vitamin A intake and serum vitamin A concentrations are significantly lower in patients with asthma than in healthy control subjects and are lower in patients with severe asthma than in those with mild asthma [36, 37]. One study shows that low vitamin A status increases susceptibility to cigarette smoke-induced lung emphysema in a mouse model [38]. However, other studies suggest that dietary supplementation with vitamins, such as vitamin A and ascorbate, does not improve lung function or asthma symptoms [39–41]. We found no relationship between overall dietary antioxidant intake and asthma diagnosis or wheeze in the previous 12 months, although children who were exposed to ETS and had a low dietary vitamin A intake were more likely to report symptoms of asthma. Dietary antioxidants may ameliorate the effects of smoking on asthma symptoms, although future human studies that assess the benefits of antioxidant intake should focus on selecting an appropriate exposure to oxidative stress.

Recent studies suggest that genetic factors may also contribute to an individual’s susceptibility to respiratory disorders induced by ETS exposure [7]. *GSTP1* encodes for an enzyme that belongs to a large family of GST enzymes, which are important for detoxification of potentially harmful compounds from tobacco smoke, such as the polyaromatic hydrocarbon molecules benzopyrene and chrysene [42, 43]. *GSTP1* is widely expressed in human airways, predominantly in alveolar macrophages and epithelial cells [44]. Polymorphisms in the GST genes, such as *GSPT1* (rs1695), affect the ability to respond to excessive oxidative stress by altering activity of the GST enzymes [45]. Based on the hypothesis that dietary intake of antioxidants and endogenous antioxidant capacity contribute to the susceptibility to oxidative stress in asthmatic children, researchers investigated the effects of antioxidant supplementation on ozone-related decreases in lung function according to *GSTM1* genotype [46, 47]. Children in the placebo group that lacked the *GSTM1* gene had a significant reduction in FEF_25–75%_ after ozone exposure, whereas the *GSTM1*-positive children in the placebo group did not. Therefore, asthmatic children with compromised antioxidant defense systems caused by genetic susceptibility and deficiencies in antioxidant intake may be at increased risk for oxidative stress induced by ozone or ETS. However, a recent meta-analysis indicates that the *GSTP1* single nucleotide polymorphism rs1695 did not affect the prevalence of asthma, suggesting that presence of GST variants contribute to airway diseases through interactions with the environment [48].

Active *GSTP1* variant proteins produced by the *GSTP1* gene play a role in xenobiotic metabolism and influence susceptibility to asthma and other diseases [49]. Some studies show that *GSTP1* encodes for an important enzyme in the anti-oxidative pathway that buffers the harmful effects of air pollution [21, 50]. The interaction between *GSTP1* and different types of air pollutants has a higher information gain than other gene-air pollutant combinations [21]. Therefore, we investigated the influence of interactions between ETS, dietary antioxidant intake, and the *GSTP1* gene on risk for childhood asthma. We also investigated the relationship between *MTHFR* (rs1801133) and *NQO1* (rs1800566) genes and asthma symptoms, but we did not present these data because we did not find an association.

To the best of our knowledge, few publications have investigated the overall effects of GST variants and ETS exposure on asthma symptoms [51–55], and whether such effects could be modulated by dietary antioxidant intake has not yet been explored. This was the first study to assess how ETS, low dietary vitamin A intake, and *GSTP1* genotype affect asthma symptoms in children. However, our study had some limitations. First, this was a cross-sectional study, and therefore we could not determine causal relationships among the factors studied. Second, we focused on only one well-known candidate gene involved in oxidative stress, and other genes likely also regulate the influence of ETS and dietary antioxidants. Third, our study may also have recall bias because our dietary data were based on the semi-quantitative FFQ completed by parents or guardians, who may have underreported unhealthy foods and overreported healthy foods. Fourth, the number of children in the asthma group was smaller than the number in the control group, a discrepancy that is common in community-based studies. In addition, we did not record the use of other supplements, such as multivitamins, and we could not confirm the association between dietary intake and serum levels of antioxidants because we did not measure serum levels. Nonetheless, the clinical implications of these findings are important because exposure to ETS is common in children. Further prospective, long-term follow-up studies are needed to confirm and extend these findings.

## Conclusion

Our data showed that low dietary intake of vitamin A and exposure to ETS may increase oxidative stress, which may increase the risk of asthma in children. These relationships may be shaped further by genetic susceptibility alleles of *GSTP1*.

## Additional files

Additional file 1: Table S1. Characteristics of the study population and subjects not included in the current analysis (DOC 43 kb) Additional file 2: Table S2. Combined effects of dietary antioxidant intake, ETS, and *glutathione S-transferase P1* (*GSTP1*) polymorphism on diagnosis of asthma (DOC 65 kb)
