# Supplementary material for: Modification of additive effect between vitamins and ETS on childhood asthma risk according to GSTP1 polymorphism : a cross -sectional study
Source: BMC Pulm Med. 2015 Oct 22;15:125. doi: 10.1186/s12890-015-0093-0 (PMC4618939; doi:10.1186/s12890-015-0093-0)
Supplement: Additional file 1: Table S1. — Characteristics of the study population and subjects not included in the current analysis (DOC 43 kb) [file 12890_2015_93_MOESM1_ESM.doc]

**Additional file 1: Table S1. Characteristics of the study population and subjects not included in the current analysis**

| Parameter | | Children included  in this study  (*n*=1111) | Children not included  in this study  (*n*=245) | P-value |
| --- | --- | --- | --- | --- |
| Age (years) | | 9.48±1.73 | 10.54±1.48 | <0.001 |
| Sex (male/female) | | 577/529 | 141/97 | 0.047 |
| BMI | | 18.46±3.30 | 18.91±3.30 | 0.046 |
| Parental history of asthma | | 38/875 (4.3%) | 2/111(1.8%) | 0.304 |
| Parental history of AR | | 347/885 (39.2%) | 40/119(33.6%) | 0.239 |
| Parental history of AD | | 88/896 (9.8%) | 6/111(5.4%) | 0.131 |
| Maternal education | Low  (≤ high school) | 391/1090 (35.9%) | 59/137(43.1%) | 0.100 |
|  | High | 699/1090 (64.1%) | 78/137(56.9%) |  |
| Household income  (10,000 Korean won) | ≤ 299 | 312/1055 (29.6%) | 50/131(38.2%) | 0.130 |
|  | 300–399 | 292/1055 (27.7%) | 31/131(23.7%) |  |
|  | ≥ 400 | 451/1055 (42.8%) | 50/131(38.2%) |  |
| Paternal smoking | Non-smoker | 293/1078 (27.2%) | 34/129(26.4%) | 0.913 |
|  | Past smoker | 263/1078 (24.4%) | 30/129(23.3%) |  |
|  | Current smoker | 522/1078 (48.4%) | 65/129(50.4%) |  |
| Maternal smoking | Non-smoker | 1075/1091 (98.5%) | 131/136(96.3%) | 0.114 |
|  | Past smoker | 6/1091 (0.6%) | 2/136(1.5%) |  |
|  | Current smoker | 10/1091 (0.9%) | 3/136(2.2%) |  |
| Environmental tobacco smoke | | 403/1111 (36.3%) | 51/121(42.2%) | 0.203 |
| Exposure frequency  of ETS (per week) | ≤ twice | 85/215 (39.5%) |  |  |
|  | 2–4 times | 59/215 (27.4%) |  |  |
|  | ≥ 5 times | 71/215 (33.0%) |  |  |
| Wheeze in previous 12 months | | 62/1069 (5.8%) | 4/129(3.1%) | 0.204 |
| Asthma diagnosis | | 110/1064 (10.3%) | 5/131(3.8%) | 0.017 |

AR, allergic rhinitis; AD, airway disease; BMI, body mass index; ETS, environmental tobacco smoke
