# Supplementary material for: Modification of additive effect between vitamins and ETS on childhood asthma risk according to GSTP1 polymorphism : a cross -sectional study
Source: BMC Pulm Med. 2015 Oct 22;15:125. doi: 10.1186/s12890-015-0093-0 (PMC4618939; doi:10.1186/s12890-015-0093-0)
Supplement: Additional file 2: Table S2. — Combined effects of dietary antioxidant intake, ETS, and glutathione S-transferase P1 (GSTP1) polymorphism on diagnosis of asthma (DOC 65 kb) [file 12890_2015_93_MOESM2_ESM.doc]

**Additional file 2: Table S2. Effects of dietary antioxidant intake, ETS, and *glutathione S-transferase P1* (*GSTP1*) polymorphism on diagnosis of** asthma

| Variable | | Genotype AA | | **Asthma diagnosis** | | | Genotype AG+GG | **Asthma diagnosis** | | | | | |
| --- | --- | --- | --- | --- | --- | --- | --- | --- | --- | --- | --- | --- | --- |
|  |  | N (%) | | aOR* (95% CI) P-value | | | N (%) | aOR* (95% CI) P-value | | | | | |
| **Vitamin A** | **ETS** |  |  |  |  |  |  |  |  |  | |  |  |
| High | No | 10/111(9.01) | 1.00 |  |  |  | 8/66(12.12) | 1.00 |  |  |  | |  |
| Low | No | 20/250(8.00) | 1.14 | (0.42 | 3.13) | 0.80 | 10/137(7.30) | 0.55 | (0.14 | 2.17) | 0.40 | |  |
| High | Yes | 6/66(9/09) | 1.44 | (0.45 | 4.64) | 0.54 | 5/50(10.00) | 0.78 | (0.20 | 3.12) | 0.73 | |  |
| Low | Yes | 22/136(16.18) | **4.44** | **(1.58** | **12.52)** | **<0.01** | 12/83(14.46) | 2.04 | (0.54 | 7.69) | 0.29 | |  |
| **Retinol** | **ETS** |  |  |  |  |  |  |  |  |  |  | |  |
| High | No | 12/124(9.68) | 1.00 |  |  |  | 9/6(13.24) | 1.00 |  |  |  | |  |
| Low | No | 18/237(7.59) | 1.05 | (0.38 | 2.87) | 0.93 | 9/135(6.67) | 0.48 | (0.12 | 1.92) | 0.30 | |  |
| High | Yes | 13/67(19.40) | **4.18** | **(1.51** | **11.57)** | **<0.01** | 9/49(18.37) | 2.16 | (0.58 | 8.10) | 0.25 | |  |
| Low | Yes | 15/135(11.11) | 2.15 | (0.77 | 6.04) | 0.15 | 8/84(9.52) | 0.84 | (0.21 | 3.35) | 0.80 | |  |
| **Carotene** | **ETS** |  |  |  |  |  |  |  |  |  |  | |  |
| High | No | 10/115(8.70) | 1.00 |  |  |  | 7/62(11.29) | 1.00 |  |  |  | |  |
| Low | No | 20/246(8.13) | 0.90 | (0.34 | 2.42) | 0.84 | 11/141(7.80) | 0.72 | (0.18 | 2.80) | 0.63 | |  |
| High | Yes | 7/63(11.11) | 1.73 | (0.55 | 5.38) | 0.34 | 6/50(12.00) | 1.23 | (0.32 | 4.78) | 0.76 | |  |
| Low | Yes | 21/139(15.11) | **3.15** | **(1.15** | **8.63)** | **0.02** | 11/83(13.25) | 1.93 | (0.50 | 7.48) | 0.34 | |  |
| **Vitamin C** | **ETS** |  |  |  |  |  |  |  |  |  |  | |  |
| High | No | 16/121(13.22) | 1.00 |  |  |  | 6/51(11.76) | 1.00 |  |  |  | |  |
| Low | No | 14/240(5.84) | 0.49 | (0.18 | 1.35) | 0.17 | 12/152(7.89) | 0.90 | (0.22 | 3.77) | 0.89 | |  |
| High | Yes | 9/63(14.29) | 1.27 | (0.44 | 3.69) | 0.66 | 6/49(12.24) | 2.41 | (0.59 | 9.93) | 0.22 | |  |
| Low | Yes | 19/139(13.67) | 2.23 | (0.85 | 5.84) | 0.10 | 11/84(13.10) | 1.54 | (0.36 | 6.55) | 0.56 | |  |
| **Vitamine E** | **ETS** |  |  |  |  |  |  |  |  |  |  | |  |
| High | No | 12/110(10.91) | 1.00 |  |  |  | 9/67(13.43) | 1.00 |  |  |  | |  |
| Low | No | 18/251(7.17) | 0.64 | (0.22 | 1.90) | 0.42 | 9/136(6.62) | 0.54 | (0.13 | 2.23) | 0.39 | |  |
| High | Yes | 10/54(18.52) | 2.73 | (0.96 | 7.79) | 0.06 | 8/55(14.55) | 1.80 | (0.53 | 6.13) | 0.35 | |  |
| Low | Yes | 18/148(!2.16) | 1.84 | (0.65 | 5.20) | 0.25 | 9/78(11.54) | 1.10 | (0.26 | 4.68) | 0.90 | |  |

^a^ aOR: Adjusted for age, sex, BMI (continuous), parental history of asthma, maternal education, household income, and log-transformed total energy intake.

aOR, Adjusted odds ratio; ETS, environmental tobacco smoke.
